# Gefitinib or lapatinib with foretinib synergistically induce a cytotoxic effect in melanoma cell lines

**DOI:** 10.18632/oncotarget.24810

**Published:** 2018-04-06

**Authors:** Ewelina Dratkiewicz, Katarzyna Pietraszek-Gremplewicz, Aleksandra Simiczyjew, Antonina Joanna Mazur, Dorota Nowak

**Affiliations:** ^1^ Department of Cell Pathology, Faculty of Biotechnology, University of Wroclaw, Wroclaw, Poland

**Keywords:** melanoma, EGFR inhibitor, MET inhibitor, foretinib, lapatinib

## Abstract

Melanoma is an aggressive cancer type with a high mortality rate and an elevated resistance to conventional treatment. Recently, promising new tools for anti-melanoma targeted therapy have emerged including inhibitors directed against frequently overexpressed receptors of growth factors implicated in the progression of this cancer. The ineffectiveness of single-targeted therapy prompted us to study the efficacy of treatment with a combination of foretinib, a MET (hepatocyte growth factor receptor) inhibitor, and gefitinib or lapatinib, EGFR (epidermal growth factor receptor) inhibitors. We observed a synergistic cytotoxic effect for the combination of foretinib and lapatinib on the viability and proliferation of the examined melanoma cell lines. This combination of inhibitors significantly decreased Akt and Erk phosphorylation, while the drugs used independently were insufficient. Additionally, after treatment with pairs of inhibitors, cells became larger, with more pronounced stress fibers and abnormally shaped nuclei. We also noticed the appearance of polyploid cells and massive enrichment in the G2/M phase. Therefore, combination treatment was much more effective against melanoma cells than a single-targeted approach. Based on our results, we conclude that both EGFR and MET receptors might be effective targets in melanoma therapy. However, variation in their levels in patients should be taken into consideration.

## INTRODUCTION

Malignant melanoma is a skin cancer originating from the neoplastic growth of pigment-producing cells (melanocytes). Although melanoma accounts for only 4% of all skin cancer cases, it has the highest mortality rate among cancer types [1]. According to estimations from 2012, in Europe, there were 100,000 new cases of melanoma, and 22,000 melanoma patients died in the same year [2]. Such poor prognosis and cancer aggressiveness are often associated with aberrations of receptor tyrosine kinases (RTK), such as EGFR (epidermal growth factor receptor) and MET (hepatocyte growth factor receptor), which lead to defective regulation of cell functions (e.g., proliferation, migration, control of cell cycle, and apoptosis) [3, 4].

EGFR, also known as ErbB1/HER1, is a member of the ErbB protein family. This group of receptors includes three additional proteins, ErbB2/HER2/Neu, ErbB3/HER3, and ErbB4/HER4. In many types of cancer, including melanoma, HER2 and EGFR are often upregulated or mutated [5]. The activity of MET in cancer cells is also frequently increased due to the amplification of the *MET* gene or its activating mutations [4]. In physiological conditions, following ligand binding, both receptors dimerize and undergo autophosphorylation which leads to activation of downstream signaling pathways. This includes pathways such as the Ras/mitogen-activated protein kinase (MAPK) or phosphatidylinositol-3-kinase (PI3K)/Akt [6]. However, a mutation in a catalytic domain of a receptor might be the cause of its constitutive phosphorylation and activation. This could result in upregulation of functions mediated by stimulated pathways, including increased cell proliferation, migration, and invasion, as well as decreased susceptibility to proapoptotic signals and impaired regulation of cell cycle [7].

Among currently used melanoma-targeted therapies is treatment based on the use of small molecule inhibitors. These inhibitors can directly target receptor tyrosine kinases or downstream proteins [8, 9]. Foretinib, the potent inhibitor of MET, VEGFR (vascular endothelial growth factor receptor), RON and AXL, which binds to receptors competitively with ATP [10], has been used as a first-line therapy in patients with hepatocellular carcinoma (phase I/II) [11], HER2-positive (phase I) [12], and triple-negative breast cancer (phase II) [13], metastatic gastric cancer (phase II) [14], and papillary renal cell carcinoma (phase II) [15]. Gefitinib (Iressa^®^) selectively inhibits autophosphorylation of EGFR and is mainly used for the treatment of chemoresistant non-small cell lung cancer (NSCLC) patients [16]. Lapatinib (Tyverb^®^) targets EGFR and HER2 and acts similarly to gefitinib by inhibiting autophosphorylation of these receptors. However, contrary to other EGFR inhibitors, lapatinib can bind to an inactive form of its target [17]. Lapatinib is often used in combination therapy with monoclonal antibodies or other small molecule agents in patients with HER2-positive metastatic breast cancer [18, 19].

Due to frequently reported abnormalities in the regulation of MET and ErbB protein expression among patients with melanoma, these receptors are promising therapeutic targets. However, monotherapies require administration of higher doses of drugs, which often leads to acquired resistance [20]. Also, there are many reports indicating crosstalk between receptor tyrosine kinases, including MET and EGFR [21]. This interaction could be responsible for amplification of signal transduction governed by these proteins and compensation of function in the case when only one of the receptors is inhibited. Hence, combined therapy targeting both receptors is required to effectively suppress activation of shared signal transducing pathways and crosstalk-induced positive feedback loops [20]. This study aimed to determine the potential combination of drugs that could be successfully used against human melanoma cells. Liu *et al.* obtained promising results using a mix of foretinib and lapatinib on a panel of human cancer cells including breast, lung, and gastric carcinoma cell lines but did not test melanoma cell lines [22]. Here, we show the synergistic effect of the combination of foretinib and lapatinib on the cytotoxicity and proliferation of melanoma cell lines characterized by different levels of RTK expression and sensitivity to small molecule inhibitors.

## RESULTS

### Expression and activation levels of the ErbB family and MET in the examined melanoma cell lines

**Figure 1:**
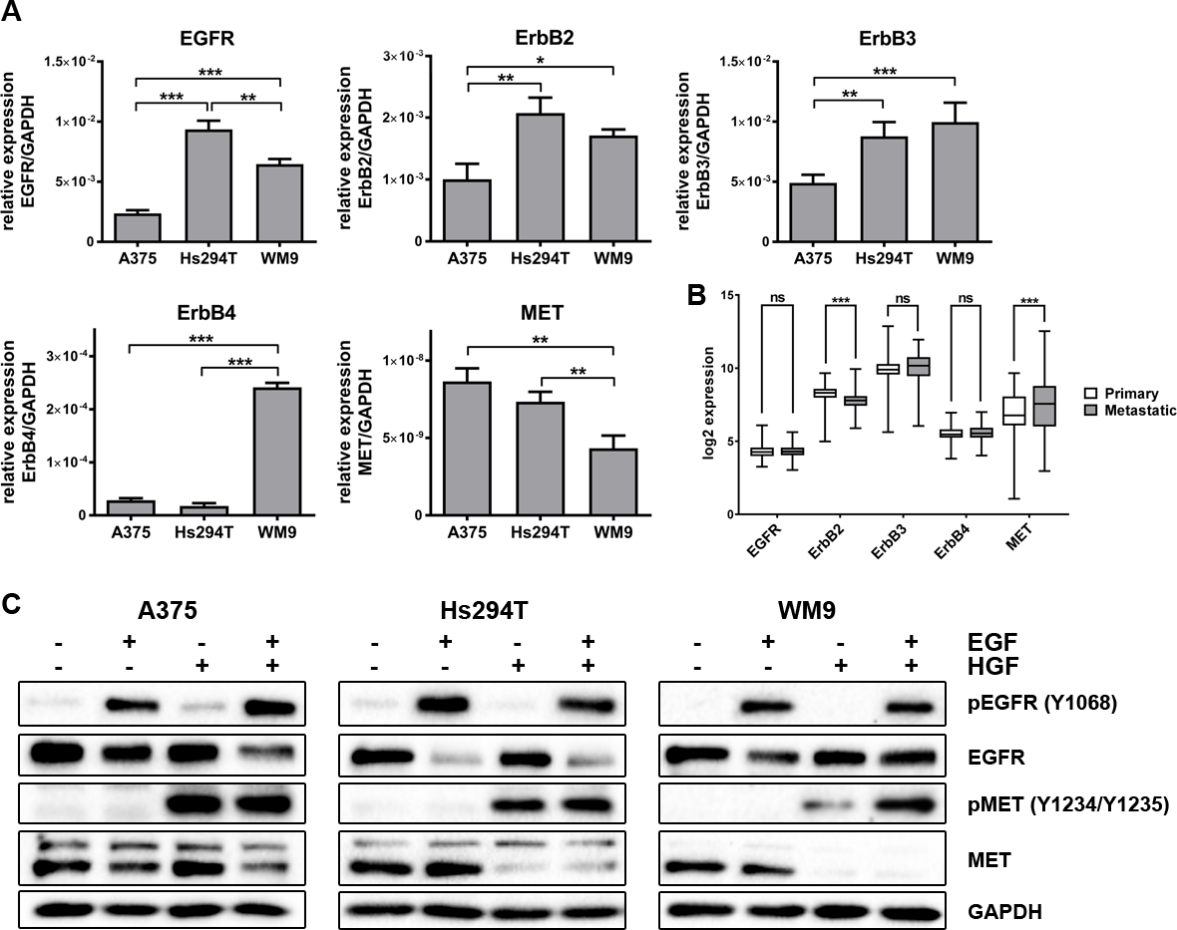
Expression level of ErbB family and MET, and activation level of selected receptors in melanoma cell lines (**A**) qRT-PCR analysis of EGFR, ErbB2, EbrB3, ErbB4 and MET levels. Results are expressed as the mean (relative gene expression compared to GAPDH) ± SD of three independent experiments. *p* ≤ 0.05 (^*^), *p* ≤ 0.01 (^**^), *p* ≤ 0.001 (^***^). (**B**) Log2 expression of selected receptors across 269 melanoma samples (114 primary and 155 metastatic) from GEO database. *p* ≤ 0.05 (^*^), *p* ≤ 0.01 (^**^), *p* ≤ 0.001 (^***^). (**C**) Western blot analysis of pEGFR/EGFR and pMET/MET in cells incubated with culture medium alone or containing EGF, HGF or both for 4 h. Membranes were probed with antibodies against total and phosphorylated EGFR and MET and are representative for at least three independent experiments. GAPDH was used as the sample loading control.

Three melanoma cell lines were chosen to conduct our studies: one isolated from primary amelanotic tumor (A375) and two derived from lymph node metastases (Hs294T and WM9). While in our previous experiments we have shown that EGFR and MET are expressed in our panel of cell lines [23], here we decided to further characterize them by estimation of expression levels of members of the ErbB family (ErbB2, ErbB3, and ErbB4). Using qRT-PCR, we detected differences in the expression of these receptors in the examined cells (Figure 1A). We noted that EGFR, ErbB2, and ErbB3 levels were increased in metastatic cell lines compared to those derived from primary tumors. The most significant diversification was observed in the case of ErbB4, where the highest expression was exhibited by WM9 cells. To gain an insight into the expression levels of these proteins among patients with melanoma, we analyzed publicly available data from gene expression microarrays deposited in the Gene Expression Omnibus (N_primary_ = 114, N_metastatic_ = 155). The first thing we noticed was that both primary and metastatic tumors showed expression of all five receptors. Therefore, we propose that these proteins can serve as targets for an anti-melanoma treatment (Figure 1B). However, only ErbB2 and MET levels were found to be statistically different between samples from primary and metastatic tumor tissue and, along with ErbB3, they exhibited the highest variation of expression values. We also examined the activation of EGFR (phosphorylated Y1068) and MET (phosphorylated Y1234/Y1235) proteins upon stimulation with growth factors (Figure 1C). These data demonstrate that the studied cell lines are responsive to growth factor treatment, as evidenced by the appearance of phosphorylated receptors.

### The effect of a combined EGFR and MET inhibitor treatment on melanoma cell viability and proliferation

In many melanoma cases, EGFR and MET are overexpressed or upregulated as a result of gene mutation [4, 5]. Combined with the fact that these receptors are involved in crosstalk and share some signaling pathways, this can lead to acquired resistance among patients treated with single inhibitor therapy [21, 24, 25]. For this reason, we decided to study the effect of an EGFR (lapatinib, gefitinib) and MET (foretinib) inhibitor combination on the viability and proliferation of selected melanoma cell lines.

**Figure 2:**
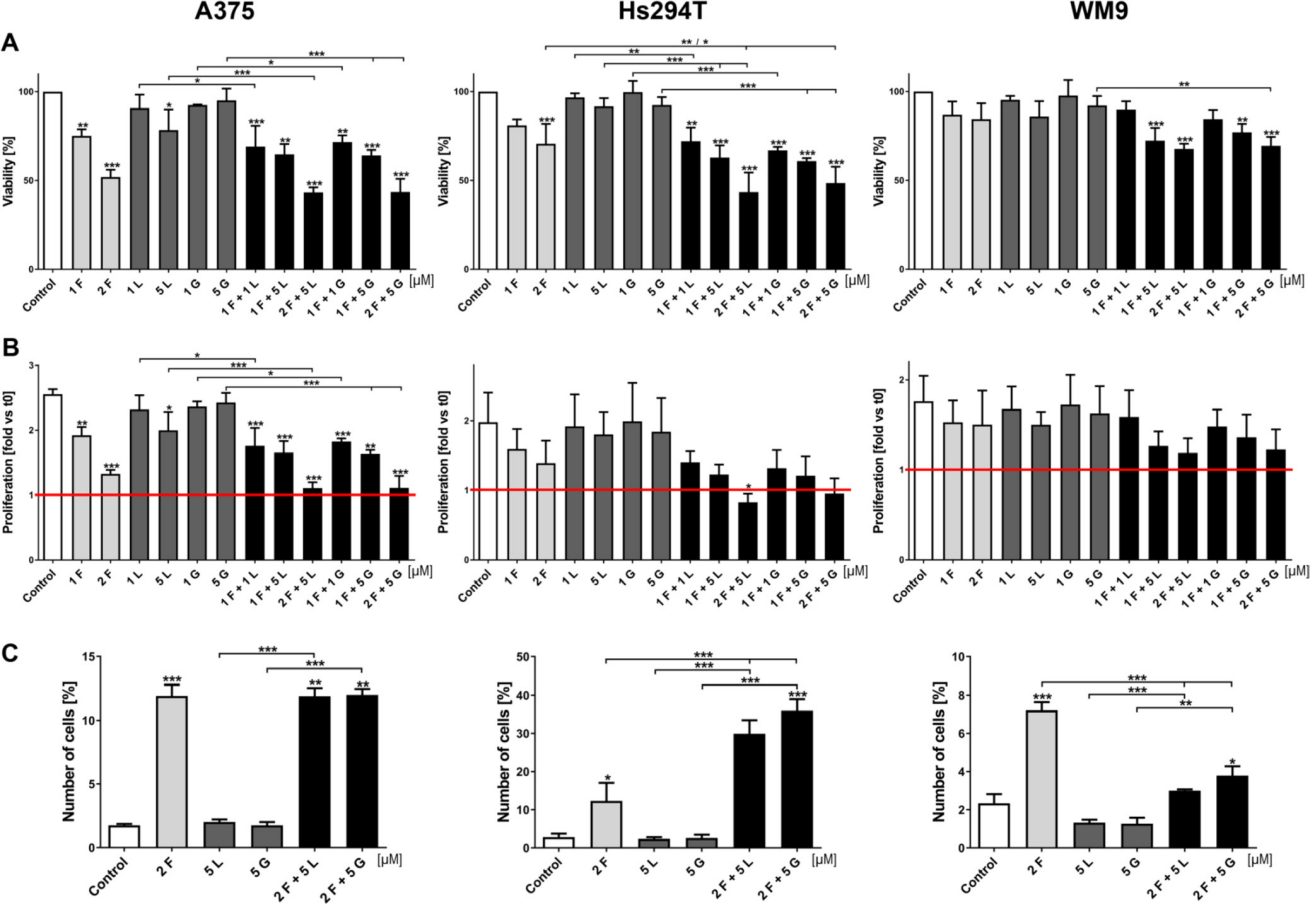
Effect of EGFR and MET inhibitors on melanoma cells viability, proliferation rate and apoptosis (**A**) Viability of melanoma cells treated for 24 h with indicated concentrations of foretinib (F), lapatinib (L) and gefitinib (G) independently and in combinations was compared to viability of control cells. Results are expressed as the mean (% of control) ± SD of three independent experiments. (**B**) Based on viability results, proliferation rate was calculated as a ratio to proliferation of untreated cells at t0. Results are expressed as the mean (fold change) ± SD of three independent experiments. Red line indicates proliferation rate of control cells at t0. (**C**) Apoptotic effect of inhibitors used independently and in combinations for 48 h. At least 30,000 cells were acquired for each sample by flow cytometry. Results are presented as a mean (percentage of cells exhibiting both markers - of early (FITC-positive, PI-negative) and late (FITC-positive, PI-positive) apoptosis) ± SD of three independent experiments. Asterisks above the bars express significance *vs*. control unless indicated otherwise. For all experiments *p* ≤ 0.05 (^*^), *p* ≤ 0.01 (^**^), *p* ≤ 0.001 (^***^).

First, we decided to test different RTKs inhibitors in a wide range of concentrations. We used erlotinib, lapatinib, and gefitinib (inhibitors of EGFR) and crizotinib and foretinib (inhibitors of MET), independently and in pairs (anti-EGFR and anti-MET drugs) to determine their influence on cell viability and proliferation (data not shown). For later studies, we chose the most promising combinations - foretinib with lapatinib or gefitinib. All three examined cell lines were treated with the indicated concentrations of EGFR (1 or 5 μM lapatinib, gefitinib) and MET (1 or 2 μM foretinib) inhibitors alone or in combination for 24 (Figure 2A and 2B) or 48 h (Supplementary Figure 1A and 1B) in the presence of EGF and HGF. To determine the percent of viable cells, the XTT cell viability assay was used. The setup of the performed assay (usage of EGF and HGF simultaneously) was fashioned to mimic the physiological conditions where both receptors (EGFR and MET) and their signaling pathways are stimulated. Data acquired from these experiments suggest that only foretinib used independently can significantly decrease the cell viability in a dose-dependent manner, except in WM9 cells where only minor decrease in viability was noted (Figure 2A and Supplementary Figure 1A). EGFR inhibitors have only a slight or no effect regardless of drug concentration, whereas combinations of foretinib with lapatinib or gefitinib decreased melanoma cell viability in a dose-dependent manner, especially in the A375 and Hs294T cell lines. The WM9 cell line proved to be the most resistant to treatment. These results are consistent with data obtained from proliferation tests (Figure 2B and Supplementary Figure 1B), where only foretinib used independently or paired with EGFR inhibitors was able to diminish the rate of proliferation; however, this reached statistical significance only for the A375 cell line. Again, the WM9 cell line was the least sensitive to inhibitor treatment. We did not observe a striking difference between combinations of EGFR inhibitors with foretinib. It is worth noting that in almost all examined cell lines, the application of pairs of inhibitors demonstrated not only additive but also synergistic effects (Table 1 and Supplementary Table 1). We also measured the proliferation rate using a method based on cell confluence rather than the metabolic activity of cells. By this approach, the inhibitor treatments produced similar trends to the XTT assay, with slight differences noticed for foretinib (Supplementary Figure 2A). Additionally, we observed an increase in the size of the studied cells after treatment with foretinib alone and when paired with EGFR inhibitors (Supplementary Figure 2B), which, together with differences in the cell size and growth rate between cell lines, might account for the observed variation in the proliferation rate.

**Table 1:** Combination index values of MET and EGFR inhibitors

24 h	A375	Hs294T	WM9
**1 F + 1 G**	0.86 ± 0.12	0.60 ± 0.04	0.93 ± 0.34
**1 F + 5 G**	0.79 ± 0.19	0.60 ± 0.20	0.65 ± 0.15
**2 F + 5 G**	0.73 ± 0.08	0.62 ± 0.02	0.85 ± 0.21
**1 F + 1 L**	0.75 ± 0.16	0.59 ± 0.03	0.99 ± 0.15
**1 F + 5 L**	0.74 ± 0.21	0.55 ± 0.25	0.72 ± 0.24
**2 F + 5 L**	0.72 ± 0.20	0.38 ± 0.17	> 1.00

Based on cytotoxicity assays, combination index (CI) values were calculated with Compusyn software according to Chou and Talalay-derived equations [74] for melanoma cells treated with MET and EGFR inhibitors for 24 h. CI values are expressed as the mean ± SD of three independent experiments. CI < 1, = 1, and > 1 represent synergistic, additive and antagonistic effects, respectively.

Next, we decided to examine the proapoptotic effect of EGFR and MET inhibitors on melanoma cells. Starting from this test, all subsequent experiments were conducted only on selected inhibitor concentrations, chosen on the basis of the XTT test findings. These data are reported as percent of early (FITC-positive, PI-negative) and late apoptotic (FITC-positive, PI-positive) cells and were determined by flow cytometry (Figure 2C). After 48 h of treatment with inhibitors, the Hs294T cell line showed the highest percent of apoptotic cells above 30% for pairs of drugs and 10% for foretinib alone. The level of apoptotic-positive A375 cells was the same for the foretinib treatment and its combinations with EGFR inhibitors. After foretinib treatment less than 8% of apoptotic WM9 cells were detected, which is in line with our data from previous experiments, indicating that this cell line is the least sensitive even for pairs of inhibitors, which results in a poor level of apoptosis.

### The influence of the EGFR and MET inhibitor combination on downstream signaling

**Figure 3:**
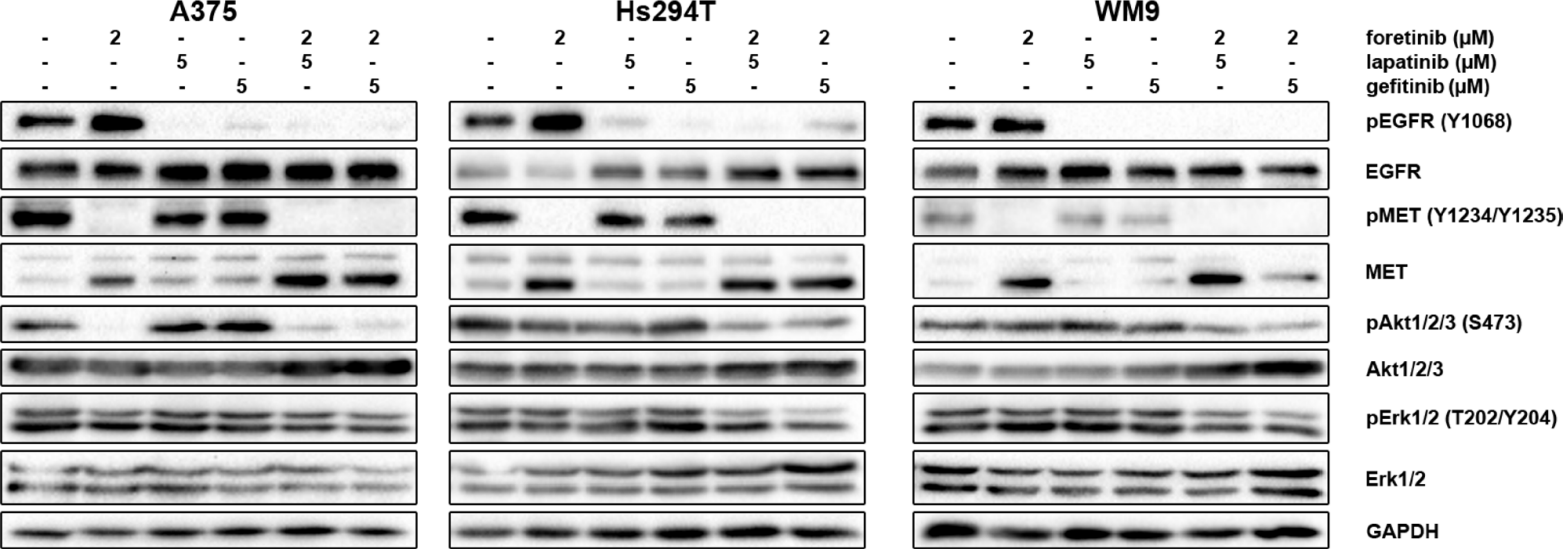
Effect of foretinib, lapatinib and gefitinib on activity of EGFR, MET, Akt, and Erk Cells were incubated with indicated concentrations of foretinib (F), lapatinib (L) and gefitinib (G) independently, and in combinations for 4 h. Membranes were probed with specific antibodies to total and phosphorylated forms of EGFR, MET, Akt, and Erk and are representative for at least three independent experiments. Detection of GAPDH serves as a loading control.

Next, we decided to investigate the effect of the tested inhibitors on the expression levels of selected proteins implicated in EGFR and MET signaling pathways in melanoma cell lines using Western blot analysis. The proteins were chosen because they are among those most frequently used as mediators in cell signaling pathways. Again, to mimic physiological conditions, cells were incubated with both EGF and HGF and the indicated concentrations of inhibitors. We found that, after stimulation of EGFR and MET all cell lines showed phosphorylation of downstream effectors (Akt and Erk) (Figure 3). Next, we tested for changes in the activation of these proteins after 4 h of incubation with selected concentrations of EGFR and MET inhibitors, independently and in pairs. We found that foretinib could diminish pMET levels and that pairing foretinib with EGFR inhibitors did not abolish this effect. Additionally, foretinib alone resulted in upregulation of the active form of EGFR, while lapatinib and gefitinib almost entirely reduced the level of pEGFR in all three cell lines. Most of the inhibitors, when used independently, were unable to affect the phosphorylation status of Akt and Erk, except in the case of A375 cells incubated with foretinib Reduced pAkt and pErk levels were observed only after the administration of pairs of inhibitors, with a more pronounced reduction of Akt phosphorylation. Additionally, the examined cell lines exhibited varying sensitivity to drug treatment, indicated by the different rates of decrease in phosphorylated proteins. Together with earlier results, these data suggest that, although single inhibitors can influence their targets by blocking phosphorylation of RTKs, in most cases, this effect is not carried over to downstream effectors and is unable to affect cell functions regulated by the targeted pathway. To alter these functions (e.g., proliferation and regulation of apoptosis), the activation of more than one receptor should be blocked.

### Effect of inhibitors on melanoma cell cytoskeleton and nucleus morphology

**Figure 4:**
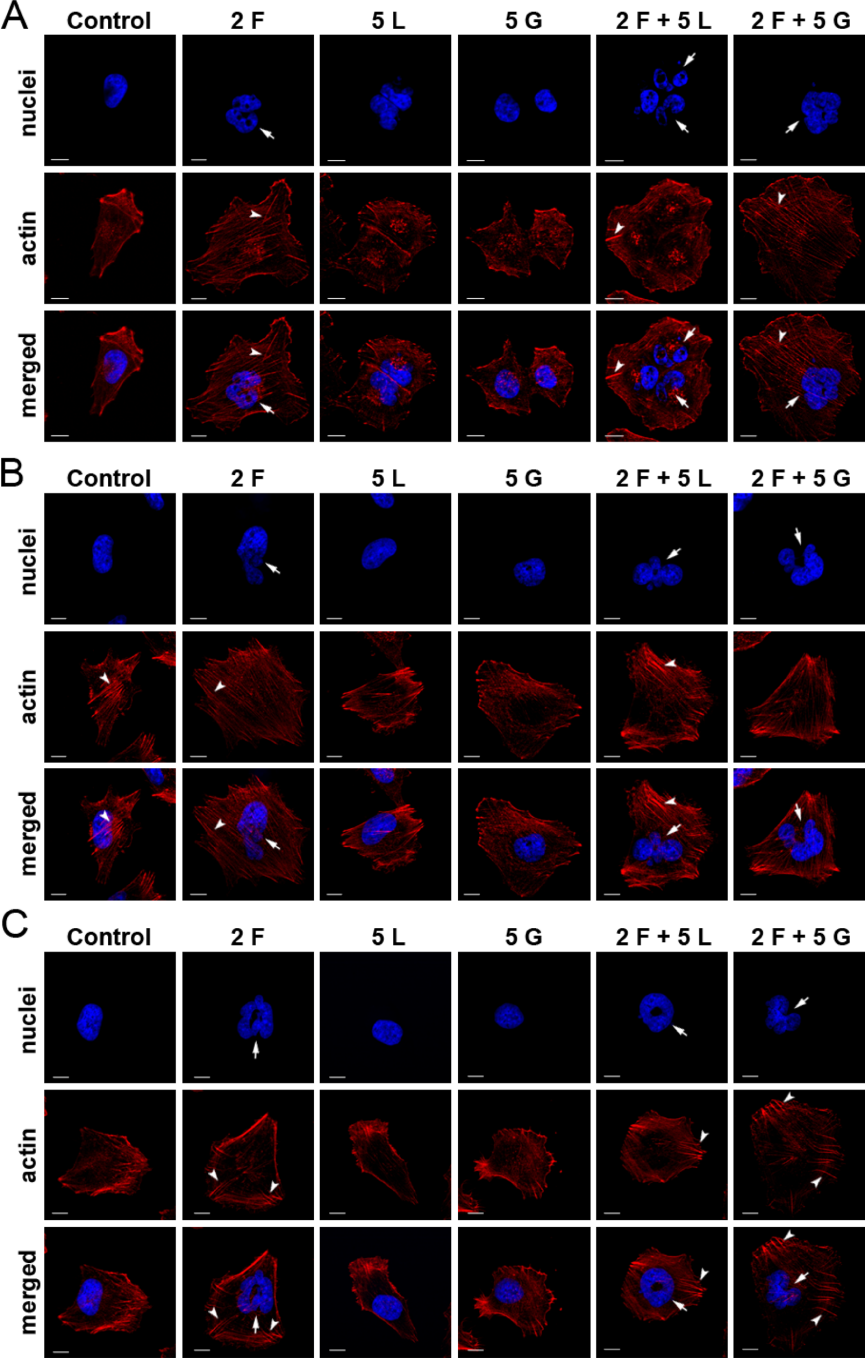
Effect of inhibitors on actin cytoskeleton organization and nuclei morphology of melanoma cells Fluorescent stainings of (**A**) A375, (**B**) Hs294T and (**C**) WM9 cells seeded on Matrigel^®^-coated coverslips, and incubated with indicated concentrations [μM] of foretinib (F), lapatinib (L), and gefitinib (G) independently, and in combinations for 24 h. Phalloidin-conjugated Alexa Fluor 568^®^ labels F-actin (red), whereas Hoechst staining (blue) was used to visualize nuclei. Arrows indicate abnormal nuclei and arrow heads - stress fibres. Scale bar represents 10 μm.

To investigate changes in cell morphology and organization, especially in the cytoskeleton, we stained melanoma cells with phalloidin conjugated with Alexa Fluor^®^ 568 (Figure 4). We observed the most substantial changes after treatment with foretinib and foretinib paired with another compound. Cells were larger and more spread on coverslips coated with Matrigel^®^ than non-treated cells and cells treated with EGFR inhibitors, which was also observed earlier in our proliferation assay (Supplementary Figure 2B). Moreover, actin stress fibers were more pronounced. Administration of lapatinib or gefitinib did not have a significant effect on cell morphology or actin cytoskeleton organization. It is worth noting that foretinib used independently or in combination also induced the dramatic change in nuclei morphology – treated cells contained multiple or larger nuclei than non-treated cells.

### Impact of foretinib on cell cycle of melanoma cells

**Figure 5:**
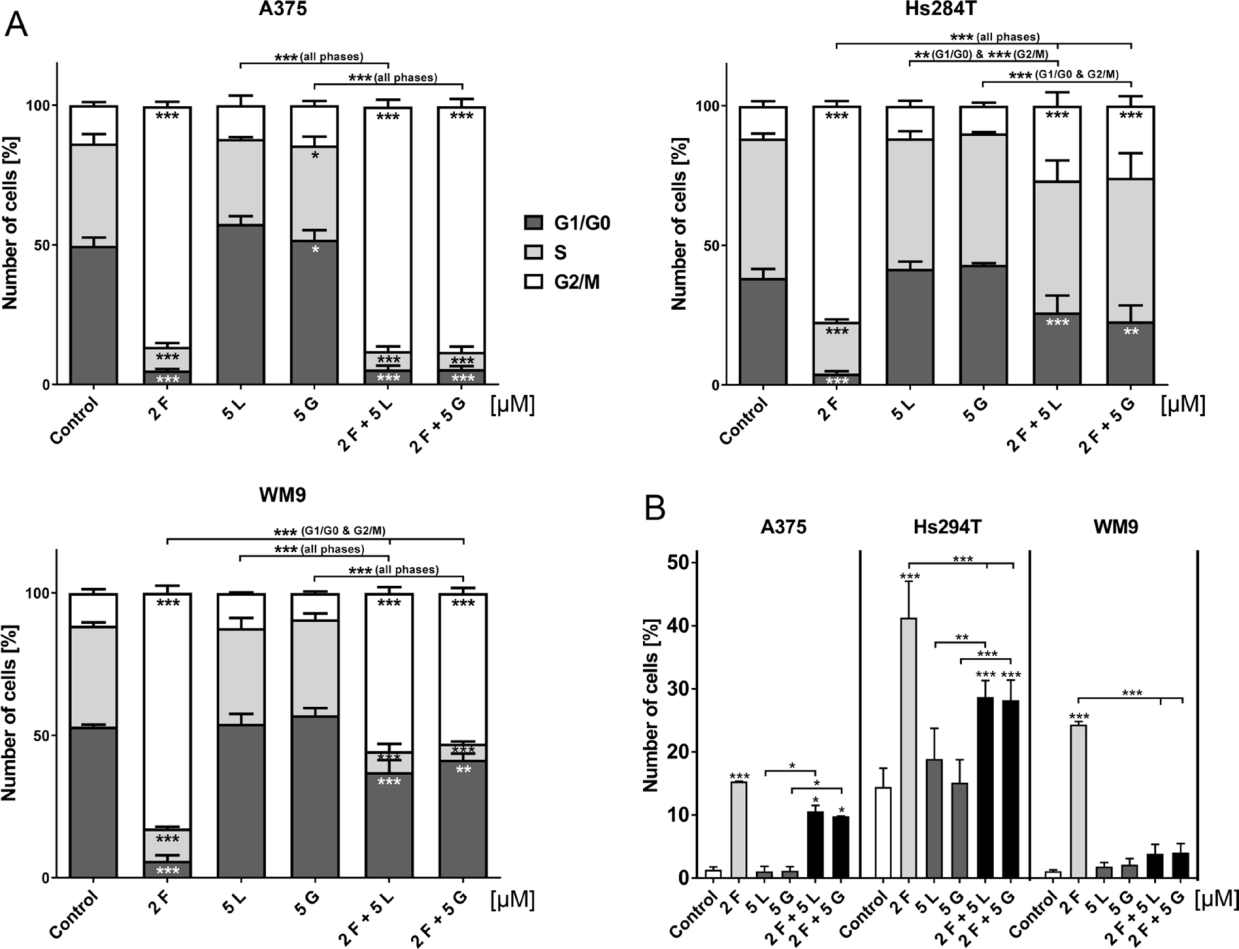
Cell cycle distribution of cells treated with EGFR and MET inhibitors (**A**) Flow cytometry analysis of apoptosis in melanoma cells incubated with indicated concentrations of foretinib (F), lapatinib (L) and gefitinib (G) independently, and in combinations for 24 h. Results are given as the mean (percentage of cell cycle) phase ± SD of three independent experiments. (**B**) Distribution of super G2 phase (above G2) in melanoma cell lines. Results are given as the mean (percentage of cell cycle) phase ± SD of three independent experiments. Asterisks below the bars express significance *vs*. control unless indicated otherwise. *p* ≤ 0.05 (^*^), *p* ≤ 0.01 (^**^), *p* ≤ 0.001 (^***^).

Due to previously observed changes in nuclei morphology upon treatment with foretinib, we decided to investigate its influence on cell cycle distribution (Figure 5A). After a 24 h treatment with the selected concentrations of EGFR and MET inhibitors (single and in pairs), we examined cell cycle distribution by flow cytometry. The cell cycle phases of all studied melanoma cell lines were drastically changed after administration of foretinib alone. For all tested cell lines, more than 70% of cells were accumulated in the G2/M phase, while the proportion of cells in the G0/G1 and S phase was extremely diminished. Treatment with lapatinib or gefitinib alone was unable to significantly change the cell cycle distribution. However, a combination of EGFR and MET inhibitors seemed to have a differential effect on the examined cell lines. While the A375 cell line treated with pairs of inhibitors exhibited almost the same pattern of cell cycle distribution as foretinib alone, both cell lines derived from metastasis (Hs294T and WM9) seemed to escape G2/M arrest caused by foretinib. We also evaluated the percentage of cells exhibiting polyploidy (an amount of DNA greater than 2N [so-called super G2 phase]) (Figure 5B). Again, we noted that cells incubated with foretinib alone showed the most significant increase in this phase. In the case of pairs of inhibitors, the data varied greatly between cell lines – for A375 and Hs294T we observed a slight decrease in the super G2 phase, while in WM9 this phase disappeared almost completely. It is worth noting that more than 15% of non-treated Hs294T cells already exhibited polyploidy.

## DISCUSSION

Melanoma represents only 4% of all cancer cases but is the most prevalent type of skin cancer and is responsible for the majority of skin cancer-related deaths [1]. While early-diagnosed primary melanoma can be successfully cured by surgical excision, metastases pose a major challenge. For that reason, the main focus of researchers is currently on the identification of molecular targets for therapies and development of congeneric treatment. Fortunately, in recent years, many experimental methods for melanoma treatment were approved for use as a first-line therapy, replacing less efficient chemotherapy. Among them are targeted molecular therapeutic approaches, immunotherapy, and the administration of oncolytic vaccines [26]. Contrary to chemotherapy, those novel types of treatment are often dependent on patients’ genotyping analyses. A prime example of such a case is the V600E mutation in the gene encoding Braf protein (a downstream effector of RTKs), which is present in 45% of diagnosed melanoma patients [26]. The United States Food and Drug Administration (FDA) and European Medicines Agency (EMA) approved two Braf V600E specific inhibitors – vemurafenib and dabrafenib – for treatment of unresectable or metastatic melanoma with mutant Braf, while a new generation of anti-Braf V600E drugs is currently in clinical trials [27]. Based on recent studies, new potential molecular targets are emerging, especially among RTKs. It has been shown that overexpression, amplification of gene copies or mutations present in the *MET* and *ErbB* genes are associated with poor diagnosis [28–30]. Those aberrations, combined with crosstalk between different RTKs [7], are responsible for the appearance of resistance and, thus, for the low efficiency of monotherapies [31]. Therefore, there is a trend to use combination therapies consisting of for example, two or more specific inhibitors of the molecular target or an inhibitor and monoclonal antibody [32]. This notion is supported by many reports concerning various cancers. Kim *et al.* observed that the MET inhibitor PHA-665752 shows a synergistic effect in combination with an EGFR inhibitor, erlotinib, in the triple negative breast cancer cell line, MDA-MB-468 [33], whereas the advantages of administrating anti-MET and anti-EGFR drugs simultaneously in non-small cell lung cancer was comprehensively reviewed by Padda *et al.* [34]. In case of melanoma management, clinical trials have focused mainly on a combination of Braf V600E and MEK inhibitors [35, 36].

In our study, we decided to focus on three inhibitors – two of which are currently used in various cancer therapies (lapatinib and gefitinib), and one is at the stage of phase II clinical trials (foretinib). According to the EMA guidelines, the daily dose of orally taken lapatinib and gefitinib for patients suffering from breast cancer and NSCLC are 1250 mg (1.35 mmol) and 250 mg (0.56 mmol), respectively. For foretinib, the daily dose is 60 mg (0.09 mmol) in case of breast cancer [13] and 240 mg (0.38 mmol) for patients with squamous cell carcinoma of the head and neck [37]. During serial administration, drug accumulation might occur, later resulting in a steady therapeutics concentration in the plasma. However, in the long term maintaining a high concentration might be followed by severe side effects. Therefore, our study aimed to design a treatment against melanoma cells using a combination of small inhibitors of RTKs, applying overall lower concentrations of the studied drugs. There is little data available on targeting multiple RTKs in this particular cancer type. Schicher *et al.* found that, in melanoma cell lines and mouse xenografts, erlotinib (an EGFR inhibitor) and bevacizumab (an anti-VEGF monoclonal antibody) act synergistically to decrease proliferation, 3D invasion, and activation of signaling pathways in their cell and animal models [38]. Other studies have reported the successful application of MET inhibitors paired with an anti-Braf V600E drug [39] and therapy including the pan-ErbB inhibitor, canertinib, and Braf V600E inhibitor [40]. In another study, Liu *et al.* successfully applied foretinib in combination with lapatinib against various cancer cell lines but no melanoma cells were tested [22].

Based on our earlier studies (data not shown), we selected inhibitors of EGFR (lapatinib and gefitinib) and MET (foretinib) and their concentrations to use in further experiments. First, we established the expression profiles of selected RTKs in the examined cell lines derived from primary tissue (A375) and from metastasis to lymph nodes (Hs294T and WM9). We also analyzed their levels in tumor tissue samples from patients suffering from melanoma (results from public database GEO). The most noticeable changes were observed in case of MET expression, where transcript levels were significantly increased in metastatic biopsies, which is in line with reports by Sierra *et al.* [41]. For EGFR, ErbB3, and ErbB4, there was almost no difference between the two sample types. However, in those cases, activating mutations rather than gene copy amplification are often responsible for the oncogenic effect. Prickett *et al.* discovered novel mutations in ErbB4 in 19% of melanoma patients [42], while Hafner *et al.* found no evidence that the *ERBB4* gene is amplified in melanoma [43]. These observations emphasize the efficacy of personalized, targeted therapy, where each patient can exhibit different expression profile of potential oncogenes.

Next, we investigated the influence of the chosen compounds alone and in combinations on the viability and proliferation of melanoma cells. We noticed that pairs of inhibitors (foretinib/gefitinib and foretinib/lapatinib) exhibited higher cytotoxic effects compared to treatment with each of the inhibitors alone. This result was confirmed by evaluation of the combination index (CI), a commonly accepted indicator of synergy [44]. The majority of the designed drug pairs showed a synergistic effect, with CI values far below 1, especially in the case of the Hs294T cell line, where the mean CI for all combinations of inhibitors was 0.56 after 24 h and 0.52 after 48 h of treatment. These values are slightly higher (but still well below 1) than those obtained by other authors for various pairs of inhibitors applied against melanoma cells (e.g., 0.37 for combination of lapatinib and vincristine in A375 and 0.37 for pair of cediranib [PDGFR and VEGFR inhibitor and PLX-4720 [anti-Braf V600E drug] in IST-Mel1) [45].

The cytotoxicity of an anti-cancer drug depends heavily on the ability of the compound to initiate apoptosis in the targeted cells. In the case of melanoma the typically applied chemotherapy often seems to be insufficient because of its high resistance to pro-apoptotic signals. Because of this, attention is drawn to small molecular inhibitors of RTKs and other new forms of treatment [46, 47]. The ability to cause cell death by the drugs used in this study has often been reported in recent years. Foretinib induced caspase-dependent apoptosis in ovarian cancer and chronic myelogenous leukemia cell lines [10, 48], while lapatinib was particularly effective in breast cancer cell lines – triple-negative as well as HER2-positive [49, 50] - and gefitinib in non-small lung cancer cells with mutated EGFR [51]. We also decided to estimate the rate of apoptosis in our panel of cell lines after 48 h of treatment with selected concentrations of inhibitors from previous assays. These data partially corroborated with preceding results – the Hs294T cell line showed the highest number of apoptotic cells, reaching more than 30% for cells incubated with pairs of inhibitors, while for the WM9 cell line this value did not exceed 8%. The assessment of viability, proliferation rate, and apoptosis level after drug administration allowed us to differentiate the examined cell lines on the basis of sensitivity to inhibitor treatments, with the WM9 cell line exhibiting the lowest sensitivity, while A375 and Hs294T showed similar (high) responsiveness.

So far, our data demonstrated the level of influence of the examined RTK inhibitors on cell survival. However, because of frequently reported cases of resistance to inhibitor treatment, it is essential to note the complexity of signal transduction that originates on the cell surface with stimulation of RTKs and continues through often shared downstream pathways. On the one hand, authors point out the importance of receptors’ crosstalk [52, 53] and coactivation [7, 54], gene copy amplification [55], and mutations [56, 57], but on the other hand, they acknowledge the impact of simultaneously activated effector proteins involved in these pathways [21]. In our study, we decided to focus on selected elements of the Ras/MAPK and PI3K/Akt pathways, namely Erk and Akt, which are the main mediators of the pro-survival/anti-apoptotic signal. After incubation with lapatinib or gefitinib and their pairs with foretinib we observed total inhibition of EGFR phosphorylation while treatment with foretinib only resulted in pEGFR upregulation compared to control cells. The situation was similar with inhibition of MET and its respective inhibitor. In case of pAkt and pErk, a significant decrease was observed only after treatment with a combination of inhibitors, with pAkt showing a higher rate of responsiveness. Liu *et al.* also reported more pronounced decrease in the rate of Akt and Erk phosphorylation after incubation with combinations of foretinib with erlotinib or lapatinib in the presence of HGF for a panel of human cancer cell lines [22]. However, in our study, constant Erk activation should not be surprising. For one, during all assays, the culture medium was supplemented with EGF and HGF, which led to simultaneous activation of MET and EGFR and subsequently also Erk. Moreover, it has been reported that Erk is phosphorylated in 70% of melanoma cases, probably due to mutated and constitutively active RTKs [58]. These results confirm the necessity of combination therapy in melanoma patients.

One of the features regulated by downstream effectors of RTKs is cytoskeleton organization. Among others, cytoskeleton organization defines cell shape, volume, and polarity, as well as regulation of chromosomal separation during cell division [59]. Therefore, in the next step, we decided to examine the potential changes in the morphology of melanoma cells after drugs administration. Substantial changes were observed only in cells treated with foretinib and its pairs with EGFR inhibitors. In phase contrast pictures, cells appeared bigger and more spread, while fluorescent staining of the actin cytoskeleton revealed more pronounced stress fibers, structures built by filamentous actin. These observations were analogous to previous reports concerning melanoma cells carrying a Braf V600E mutation treated with vemurafenib [60] and human umbilical vein endothelial cells treated with FAK inhibitors [61]. Although orchestrated by several signaling pathways, actin cytoskeleton remodeling [62] underlies cell motility triggered by diverse motogenic external stimuli [23, 63], whereas motile structures formation is precisely controlled only by one group of small GTPases, called the Rho family. Rho proteins - transducers of various signaling pathways [64] - can be divided into three canonical types (Rho, Cdc42, and Rac). These proteins are regulated by numerous guanine nucleotide factors (GEFs), GTPase-activating proteins (GAPs), and guanine-nucleotide-dissociation inhibitors (GDIs) [65]. Activation of proper Rho proteins leads to the formation of stress fibers, whereas activated Rac and Cdc42 proteins are responsible for the occurrence of lamellipodia and filopodia, respectively [66]. Upon ligand binding RTKs, such as c-Met or EGFR pass the signal to these small GTPases via at least two ways, PI3K [66] and SOS [65] pathways. SOS, which is a classical GEF, activates Rac [65] and Ras proteins [67]. Moreover, both Ras and Rac proteins can activate PI3K [68]. Taking into consideration that the Rho family comprises over 20 members, which are controlled by vast regulators, it becomes clear that alterations in signaling pathways can lead to disturbances of the subtle balance between Rho protein family members. For instance, in canine kidney cells, it was reported that decreased Rac activity by constitutively active Ras protein resulted in increased Rho action [69]. On the other hand, results from other studies show that, due to active Rac, reactive oxygen species are generated, which downregulate Rho activity resulting in stress fibers dismantle [70]. Taken together, it seems plausible that the occurrence of stress fibers upon inhibitor treatment, as observed in this study, could be caused by a shift in the Ras/Rho/Rac balance towards more active Rho proteins. Our thesis is corroborated by the fact that we have recently shown that administration of EGF and HGF on melanoma cells causes a decrease in the filamentous to monomeric actin ratio, what was correlated with cells’ increased invasive potential [23].

Fluorescent staining analysis also revealed that cells treated with foretinib and its combinations with EGFR inhibitors exhibited abnormally shaped nuclei, or were multinucleated. To further understand the influence foretinib has on the cell nuclei we also investigated the distribution of cell cycle phases. Cells treated with foretinib and its combinations with lapatinib or gefitinib exhibited massive enrichment in the G2/M phase (G2/M arrest) in comparison to control cells and cells incubated with lapatinib or gefitinib only. Additionally, we noticed the appearance of polyploid cells with DNA content exceeding 2N. Similar results were reported for ovarian cancer cells [48] and chronic myelogenous leukemia [10]. Dufies *et al.* also noted anomalies in spindle assembly checkpoint that, with previously mentioned aberrations, evoked mitotic catastrophe [10].

In summary, our studies indicate the potential application of MET and EGFR inhibitor combinations in targeted therapy. This lead us to select pairs of gefitinib and lapatinib with foretinib that, in the specified concentrations, exhibited a synergistic effect on the viability and proliferation rate of melanoma cells what could be helpful in avoiding drug resistance. Our future aim is to examine those treatment conditions on patients’ biopsy samples derived from primary and metastatic melanoma. Our research indicates the importance of personalized treatment of patients with melanoma since we have identified the EGFR and MET as potential therapeutic targets.

## MATERIALS AND METHODS

### Cell culture and reagents

The human melanoma A375 (primary) and Hs294T (metastatic) cell lines were purchased from the American Type Culture Collection (ATCC), while WM9 cell line (metastatic) was a kind gift of Prof. Andrzej Mackiewicz from Greater Poland Cancer Center in Poznan, Poland. This cell line is available to obtain from Rockland Immunochemicals, Inc. All cell lines were authenticated by LGC Standards. Cells were grown in DMEM medium with lowered NaHCO_3_ (IITD PAN, Wrocław, Poland) containing 10% FBS, 2mM glutamine and antibiotics (100 U/ml penicillin, 100 μg/ml streptomycin) (Invitrogen). Cells were cultured in 25 cm^2^ tissue culture flasks (Sarstedt) at 37° C in 5%CO_2_/95% humidified air and passaged twice a week using 0.25% trypsin/0.05% EDTA solution (IITD PAN, Wrocław, Poland).

### Data preprocessing of microarray gene expression

Gene expression data of melanoma on three Affymetrix human genome microarray platforms (U133A, U133A2, U133APlus2) were obtained from Gene Expression Omnibus (GEO). The datasets included: GSE3189 (*N* = 45), GSE4587 (*N* = 9), GSE8401 (*N* = 83), GSE12627 (*N* = 44), GSE46517 (*N* = 83), GSE62837 (*N* = 5). Each data set was background corrected and normalized using Robust Multichip Average algorithm (RMA) and subsequently compiled and adjusted for batch effect using ComBat [71]. Probes for EGFR, ErbB2, ErbB3, ErbB4 and MET were selected with Jetset algorithm [72]. Preprocessed data consisted of 114 samples from primary and 155 from metastatic biopsies.

### Treatment of cells with inhibitors

Human recombinant epidermal growth factor (EGF) and Matrigel^®^ were purchased from BD Biosciences while human recombinant hepatocyte growth factor (HGF) was obtained from Sigma-Aldrich. Foretinib was purchased from Santa Cruz Biotechnologies and lapatinib, and gefitinib from Selleckchem. Cells were incubated with EGFR inhibitors – lapatinib and gefitinib, and/or MET inhibitor – foretinib separately or as a mix. Concentrations of inhibitors used in all assays were selected based on XTT experiments and matched to the sensitivity of a given cell line. For apoptosis assay, Western blot, fluorescent staining etc. cells were treated with foretinib (2 μM) or lapatinib (5 μM) or gefitinib (5 μM) or combination of inhibitors (2 μM foretinib with 5 μM lapatinib/gefitinib). Each time cells were stimulated with 5 nM EGF and/or 30 ng/ml HGF to imitate conditions present in melanoma microenvironment. Cells incubated only with growth factors with addition of 0.1% DMSO (solvent of inhibitors) (Sigma-Aldrich) were treated as a control.

### qRT-PCR analysis of gene expression

Total RNA was isolated from cells using GenElute^TM^ Mammalian Total RNA Miniprep Kit (Sigma-Aldrich) following the manufacturer's protocol. Concentration and quality of RNA was determined by measuring the absorbance at 260 nm and 280 nm. RNA samples, after DNase I (Sigma-Aldrich) treatment according to the manufacturer's instructions, were used for the reverse transcription reaction. cDNA template was synthesized from 1 μg of total RNA using the High Capacity cDNA Reverse Transcription Kit (Applied Biosystems). The obtained cDNA was diluted 10 times in water and quantitative PCR (qPCR) was performed using StepOne Plus Real-Time PCR (Applied Biosystems) in a mixture containing TaqMan^®^ Universal Master Mix II (Applied Biosystems), 20 ng of cDNA and specific probes in a total volume of 10 μl. The following TaqMan^®^ probes were used: GAPDH (Hs02758991-g1), EGFR (Hs01076091-m1), MET (Hs01565576-m1), ERBB2 (Hs01001580-m1), ERBB3 (Hs00176538-m1) and ERBB4 (Hs00955525-m1) (Applied Biosystems). GAPDH (glyceraldehyde 3-phosphate dehydrogenase) served as a housekeeping gene. Relative quantification of gene expression was calculated based on the comparative C_T_ (threshold cycle value) method (ΔC_T_ = C_T gene of interest_ – C_T housekeeping gene_). Three independent experiments were conducted for all cell lines.

### Cytotoxicity and proliferation evaluation

Cell Proliferation Kit II (XTT) (Roche), a colorimetric assay used to assess cell number based on their metabolic activity, was used according to the manufacturer's protocol. The XTT labeling mixture was added in parallel samples at 0 h (t0) and after 24 h (t24) or 48 h (t48) of cell growth in the presence of inhibitors. Absorbance was measured at 3 h after XTT addition and obtained values were background corrected. The mean cell viability at t24 and t48 was expressed as decrease in percentage of viability (absorbance) *vs.* control, non-treated, cells at given time point (100% of viability). For calculation of cell proliferation additional plate, measured with XTT labeling mixture at t0 (without inhibitors, start of incubation of t24 and t48 plates), was used. The mean proliferation rate was then expressed as a ratio (fold increase) at 24 h or 48 h of cell growth in the presence of inhibitors *vs.* t0. All conditions were performed in four replicates and for each cell line three independent experiments were conducted. Exact protocol of seeding cells as well as execution of test and cytotoxicity and proliferation rate calculation was described earlier by Huyck *et al.* [73].

IncuCyte Zoom System (Essen Biosciences) was applied as an alternative method to measure proliferation rate based on cell confluence. Briefly, 24 h after cell seeding medium was replaced with the fresh one and cells were incubated with selected concentrations of inhibitors for 48 h. Data was analyzed with IncuCyte ZOOM 2016A software (Essen Biosciences) and the mean proliferation was expressed as a ratio (fold increase) *vs*. t0 (the start of incubation with inhibitors). All conditions were performed in four replicates and for each cell line three independent experiments were conducted.

### Calculation of combination index

Combination analysis of treatment with foretinib and lapatinib/gefitinib (from three independent experiments) was performed using the Compusyn software program (Biosoft, Cambridge, UK) that calculates a combination index (CI) according to Chou and Talalay-derived equations [74]. CI < 1, = 1, and > 1 represent synergistic, additive and antagonistic effects, respectively.

### Apoptosis assay

24 h after cell seeding the culture medium was replaced for 48 h with the fresh one, containing indicated previously concentrations of inhibitors. Afterwards, medium was discarded, cells were washed with PBS without Ca^2+^/Mg^2+^, trypsinized, centrifuged (1000 rpm, 5 min) and incubated with FITC-conjugated Annexin V (BD Biosciences), and propidium iodide (Sigma-Aldrich) for 30 min in room temperature according to manufacturer's protocol. Subsequently, cells were analyzed with NovoCyte flow cytometer (ACEA Biosciences) and ACEA NovoExpress software (ver. 1.2.4, ACEA Biosciences). At least 30,000 gated cells (excluding debris) were acquired for each sample. Only early (FITC-positive, PI-negative) and late apoptotic cells (FITC-positive, PI-positive) were further analyzed. At least three independent experiments were performed for each cell line.

### Western blot analysis

24 h after cells seeding medium was replaced with the fresh one and cells were incubated with previously indicated concentrations of inhibitors for 4 h. Cell lysates were harvested by addition of urea buffer (50 mM TRIS-HCl pH 7.4, 5% SDS, 8.6% sucrose, 1 mM DTT, 0.45% urea), supplemented with protease and phosphatase inhibitors cocktails (Sigma-Aldrich). Protein concentration was determined with standard Bradford procedure (Sigma-Aldrich) [75]. Samples of an identical amount of protein (30 μg) were separated by 10% polyacrylamide gel electrophoresis in the presence of sodium dodecylsulfate (SDS-PAGE) according to Laemmli [76] and then transferred to nitrocellulose sheets, according to Towbin *et al.* [77]. Antibodies to EGFR (SC-03), MET (SC-10), Akt1/2/3 (SC-8312) and phospho-Akt1/2/3 (S473; SC-135651) were purchased from Santa Cruz Biotechnologies. The phospho-EGFR (Y1069; 3777), phospho-MET (Y1234/Y1235; 3077), Erk1/2 (9102), phospho-Erk1/2 (T202/Y204; 9101) antibodies were purchased from Cell Signaling Technologies. Goat anti-mouse or goat anti-rabbit antibodies conjugated with horseradish peroxidase (Cell Signaling Technologies) were applied according to the manufacturer's protocols. Immunoblots were developed using the Clarity Western ECL Substrate (Bio-Rad), scanned with ChemiDoc (Bio-Rad) and analysed with ImageLab software (ver. 6.0, Bio-Rad). At least three independent experiments were conducted.

### Fluorescent staining

The subcellular distribution of actin filaments in melanoma cells was examined by fluorescent staining. Cells were seeded on Matrigel^®^ (1 mg/ml) coated coverslips in 24-well plates. After 24 h the growth medium was replaced with the fresh one, containing previously indicated concentrations of inhibitors. Following 24 h of incubation, cells were fixed with 4% formaldehyde (Sigma-Aldrich) for 20 min at room temperature and permeabilized for 6 min with 0.1% Triton X-100 (Sigma-Aldrich) in PBS. Next, coverslips were blocked for 30 min with 1% bovine serum albumin in PBS. Actin filaments were stained with Alexa Fluor^®^ 568-labeled phalloidin and nuclei with Hoechst 33342 (Invitrogen). Coverslips were mounted with Dako fluorescent mounting medium (Dako). In each case, about 30 cells were photographed in three independent experiments (10 per repetition) and representative cells are shown. Zeiss confocal laser scanning microscope and ZEN software were used (Zeiss, ver. 12.0.0.201).

### Cell cycle analysis

24 h after cell seeding and subsequent 24 h incubation with previously mentioned concentrations of inhibitors, cells were washed with PBS without Ca^2+^/Mg^2+^, trypsinized, centrifuged (1000 rpm, 5 min) and fixed with ice-cold 70% ethanol for at least 24 h in −20° C. Then, cells were washed 3 times with PBS (2300 rpm, 5 min), incubated with RNase A (10 μg/ml, 45 min, room temperature), stained with propidium iodide (50 μg/ml, 30 min, 4° C) and subsequently analyzed with NovoCyte flow cytometer (ACEA) and ACEA NovoExpress software (ver. 1.2.4, ACEA Biosciences). At least 10,000 cells gated for singlets were acquired for each sample. At least three independent experiments were performed for each cell line.

### Statistical analysis

All data (excluding microarray analysis) are given as means ± standard deviations (SD), and their significance was determined with one-way ANOVA followed by Bonferroni post-hoc test. Gene expression data from bioinformatical analysis are presented as a median (with hinges at 25 and 75 percentiles and whiskers from the smallest to the largest value) and were assessed with the same statistical tests as other results. The significance level was set at *p* ≤ 0.05 (^*^), *p* ≤ 0.01 (^**^), *p* ≤ 0.001 (^***^). All statistical analyses were performed using GraphPad Prism software (ver. 7.03).

## SUPPLEMENTARY MATERIALS FIGURES AND TABLES


