# Supplementary material for: Gefitinib or lapatinib with foretinib synergistically induce a cytotoxic effect in melanoma cell lines
Source: Oncotarget. 2018 Apr 6;9(26):18254–68. doi: 10.18632/oncotarget.24810 (PMC5915070; doi:10.18632/oncotarget.24810)
Supplement: Supplementary file 1 [file oncotarget-09-18254-s001.pdf]

Gefitinib or lapatinib with foretinib synergistically induce a cytotoxic effect in melanoma cell lines

SUPPLEMENTARY MATERIALS

Supplementary Table 1: Combination index values of MET and EGFR inhibitors

48 h	A375	Hs294T	WM9
**1 F + 1 G**	> 1.00	0.58 ± 0.20	0.93 ± 0.15
**1 F + 5 G**	> 1.00	0.64 ± 0.24	0.94 ± 0.49
**2 F + 5 G**	> 1.00	0.59 ± 0.15	> 1.00
**1 F + 1 L**	0.98 ± 0.03	0.71 ± 0.00	> 1.00
**1 F + 5 L**	0.63 ± 0.13	0.35 ± 0.05	> 1.00
**2 F + 5 L**	0.71 ± 0.12	0.26 ± 0.06	> 1.00

Based on cytotoxicity assays, combination index (CI) values were calculated with Compusyn software according to Chou and Talalay-derived equations [64] for melanoma cells treated with MET and EGFR inhibitors for 48 h. CI values are expressed as the mean ± SD of three independent experiments. CI < 1, = 1, and > 1 represent synergistic, additive and antagonistic effects, respectively.

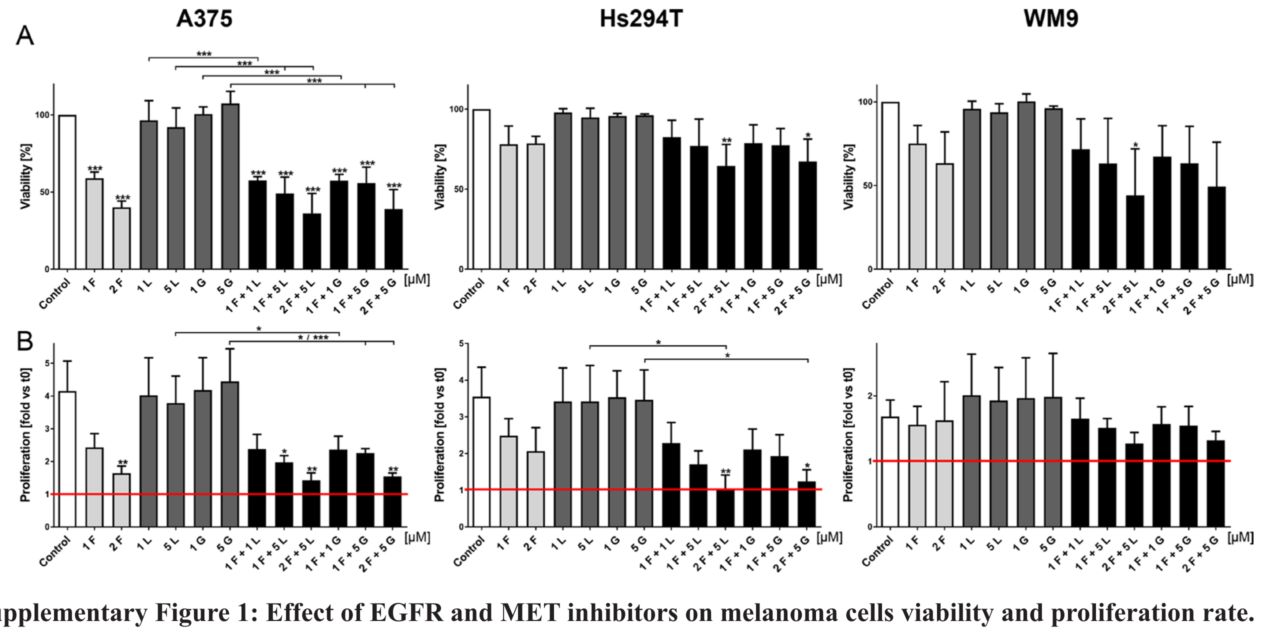

Supplementary Figure 1: Effect of EGFR and MET inhibitors on melanoma cells viability and proliferation rate. (**A**) Viability of A375, Hs294T and WM9 cells treated for 48 h with indicated concentrations of foretinib (F), lapatinib (L) and gefitinib (G) independently and in combinations was compared to viability of control cells. Results are expressed as the mean (% of control) ± SD of three independent experiments. (**B**) Based on viability results proliferation rate was calculated as a ratio to proliferation of untreated cells at t0. Results are expressed as the mean (fold change) ± SD of three independent experiments. Red line indicates proliferation rate of control cells at t0. Asterisks above the bars express significance *vs*. control unless indicated otherwise. $p \leq 0.05$ (*), $p \leq 0.01$ (**), $p \leq 0.001$ (***).

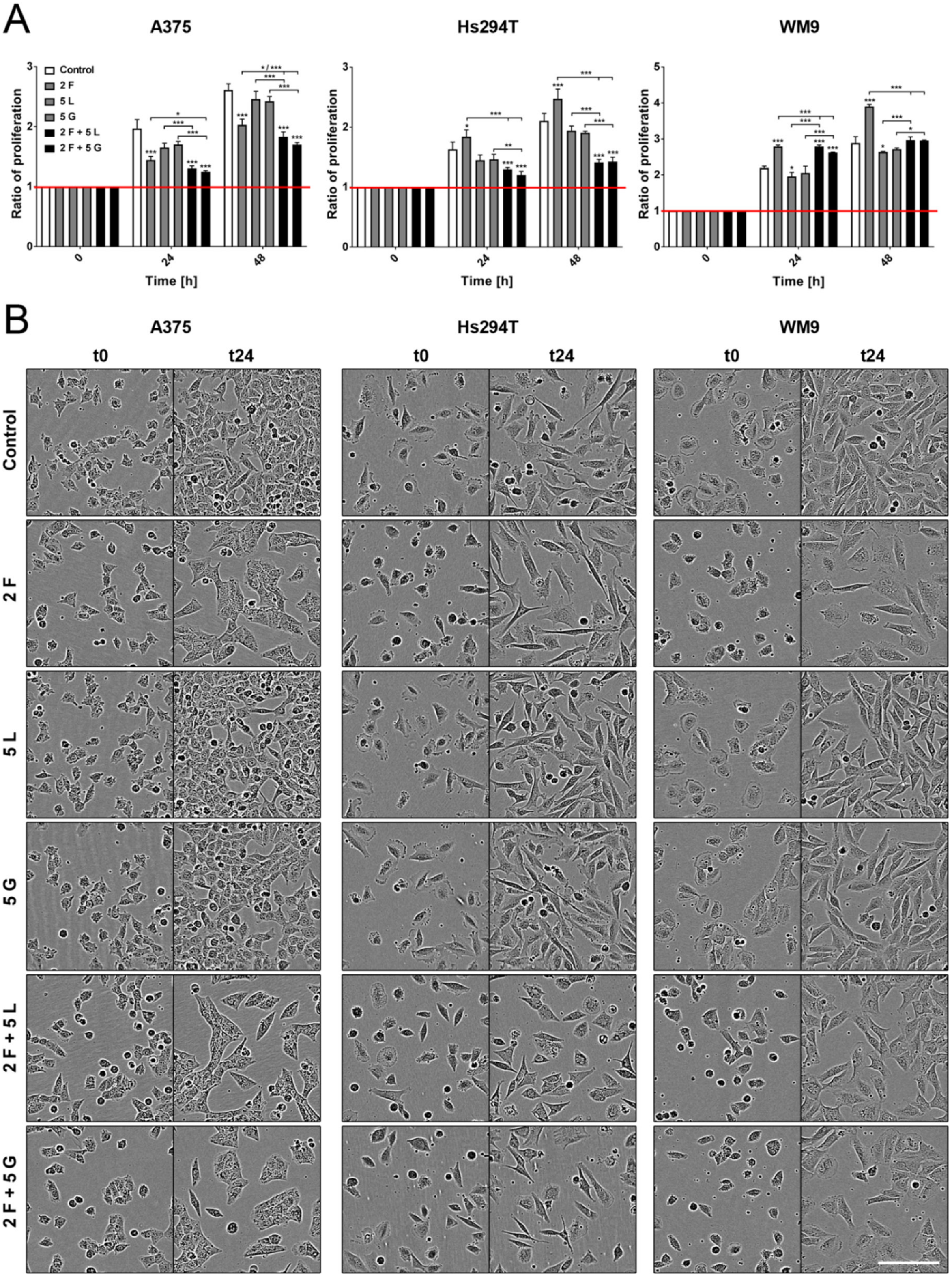

Supplementary Figure 2: Effect of EGFR and MET inhibitors on melanoma cells proliferation rate calculated based on cell confluence. (**A**) Proliferation rate of A375, Hs294T and WM9 cells treated for 48 h with selected few concentrations of foretinib (F), lapatinib (L) and gefitinib (G) independently and in combinations was calculated based on confluence. Results are expressed as the mean (fold change *vs*. t0) ± SD of three independent experiments. Asterisks above the bars express significance *vs*. control unless indicated otherwise. $p \leq 0.05$ (*), $p \leq 0.01$ (**), $p \leq 0.001$ (***). (**B**) Representative phase contrast pictures of melanoma cells from A) at t0 and t24. Scale bar is shared for all pictures and represents 150 µm.
